# Restrictive Lung Disease in Patients With Subclinical Coronavirus Infection: Are We Bracing Ourselves for Devastating Sequelae?

**DOI:** 10.7759/cureus.12501

**Published:** 2021-01-05

**Authors:** Rahul Dadhwal, Munish Sharma, Salim Surani

**Affiliations:** 1 Pulmonary Medicine, Corpus Christi Medical Center, Corpus Christi, USA; 2 Internal Medicine, Corpus Christi Medical Center, Corpus Christi, USA; 3 Internal Medicine, University of North Texas, Dallas, USA

**Keywords:** covid-19 infection, interstitial lung disease, restrictive lung disease, ground-glass opacities, angiotensin-converting enzyme 2, pulmonary fibrosis, post-covid sequelae, sars-cov-2

## Abstract

The coronavirus disease 2019 (COVID-19) pandemic has affected millions of people worldwide. The manifestations of COVID-19 infection can range from being asymptomatic to developing severe acute respiratory distress syndrome (ARDS). Here, we present a case series of five patients who were either asymptomatic or had very mild symptoms of COVID-19 infection upon diagnosis. These patients neither required a visit to the emergency department (ED) nor did they need to be hospitalized but became symptomatic and were found to have interstitial lung disease four to eight weeks after a COVID-19 diagnosis. Thus, it is imperative that we routinely follow up patients with a subclinical COVID 19 infection besides those who were symptomatic. We may be witnessing a silent surge and new-onset interstitial lung disease (ILD) as sequelae of COVID 19 infection.

## Introduction

In December 2019, a novel coronavirus was recognized to be the cause of the agglomeration of pneumonia cases in Wuhan city located in the Hubei province of China, which rapidly spread, resulting in a global pandemic [1]. There have been more than 70-million confirmed cases of COVID-19 and more than 1.6 million deaths worldwide by mid-December 2020 [2] The novel coronavirus was named severe acute respiratory syndrome coronavirus-2 (SARS-CoV-2, 2019-nCoV) due to its high parity with SARS-CoV, which caused acute respiratory distress syndrome (ARDS) and high mortality during 2002-2003 [3]. SARS-CoV-2 can spread from person to person either through droplets or via contact, and the incubation period can be up to 14 days [4]. Patients are either hospitalized or managed in the outpatient setting depending on the severity of the disease. It is a well-known fact that a significant number of patients with severe COVID-19 disease who were admitted to the hospital with respiratory symptoms had some degree of restrictive lung disease accompanied by lung scarring and fibrosis [5-6]. We hereby present five cases of patients who were asymptomatic or minimally symptomatic at the time of diagnosis of COVID-19 infection and were never admitted to a hospital nor had visited an emergency department. They presented to our pulmonary clinic after around four weeks on an average with shortness of breath and were found to have restrictive lung disease.

## Case presentation

Case 1

**Figure 1:**
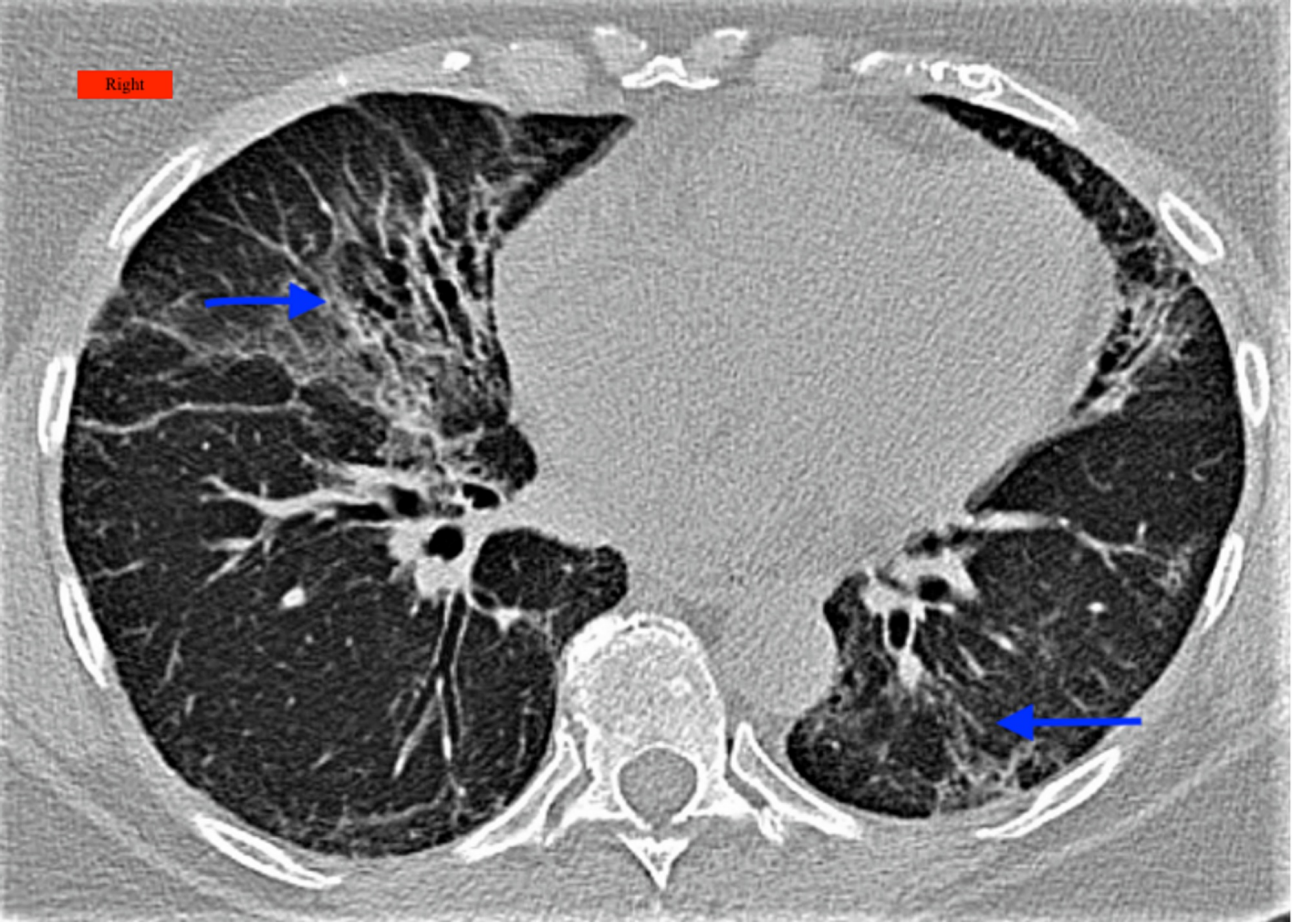
Computed tomography chest showing interlobular and intralobular septal thickening and some ground-glass opacities (arrows) Right: Indicates the right side of the patient

A 70-year-old female with a past medical history of hypertension and hyperlipidemia presented to the pulmonary clinic with complaints of shortness of breath, generalized body ache, and intermittent dry cough. The patient had fatigue and dry cough and was diagnosed with COVID-19 infection six weeks prior to the office visit. She underwent an outpatient computed tomography (CT) scan of the chest, which showed bilateral ground-glass opacities (GGOs) with intraparenchymal traction bronchiectasis. The patient received azithromycin and a methylprednisolone dose pack by her primary care physician. During the period of infection, she neither required an ED visit nor hospitalization. In the clinic, she walked 550 meters without evidence of desaturation in the six-minute walk test (6MWT). A pulmonary function test (PFT) was done, which showed a restrictive pattern (Table 1), and a repeat CT scan of the chest was done, which showed nonspecific chronic interstitial disease with areas of pulmonary fibrosis with interval improved areas of patchy GGO bilaterally (Figure 1). The patient was recommended to follow up with a repeat PFT and CT scan of the chest in three months.

**Table 1:** Table depicting pulmonary function tests of the patients after recovery from COVID infection FVC: Forced Vital Capacity; FEV1: Forced Expiratory Volume in 1 Second; TLC: Total Lung Capacity; DLCO: Diffusing Capacity for Carbon Monoxide; F: Female; M: Male

Serial Number	Age	Sex	FVC (%)	FEV1 (%)	FEV1/FVC (%)	TLC (%)	DLCO (%)
1	70	F	89	94	104	71	67
2	67	M	73	80	109	68	62
3	59	M	71	73	102	76	97
4	78	M	72	77	105	78	71
5	72	M	85	94	110	70	53

Case 2

**Figure 2:**
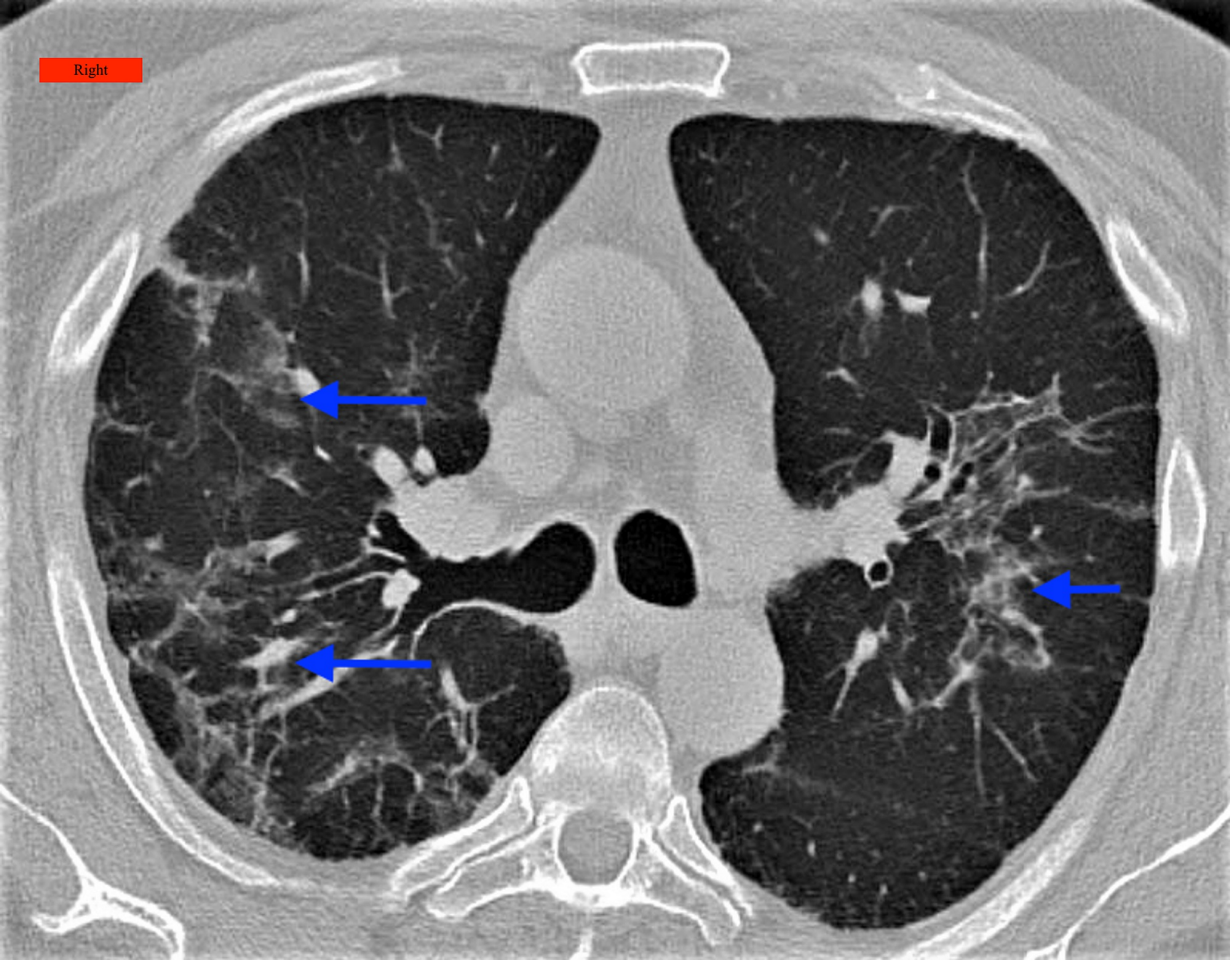
A chest CT showed areas of bilateral diffuse ground-glass opacities, interstitial thickening, and bronchial dilatation (arrows) Right: Indicates the right side of the patient; CT: Computed Tomography

A 67-year-old male, with a past medical history of hypertension, presented to the pulmonary clinic after being diagnosed with COVID-19 infection a month prior to the visit. The patient reported fatigue and shortness of breath on exertion. PFT was suggestive of a restrictive pattern (Table 1) and a chest X-ray showed patchy interstitial opacities bilaterally, with the right being worse than the left with the component of atelectasis or scarring with some linear peripheral opacities in the left mid and lower lung fields. A chest CT showed areas of bilateral diffuse GGO, interstitial thickening, and bronchial dilatation (Figure 2). The patient passed the 6MWT after walking 575 meters without desaturation and was recommended a short interval follow-up within two to three months with PFT and CT scan of the chest to assess for interval progress.

Case 3

**Figure 3:**
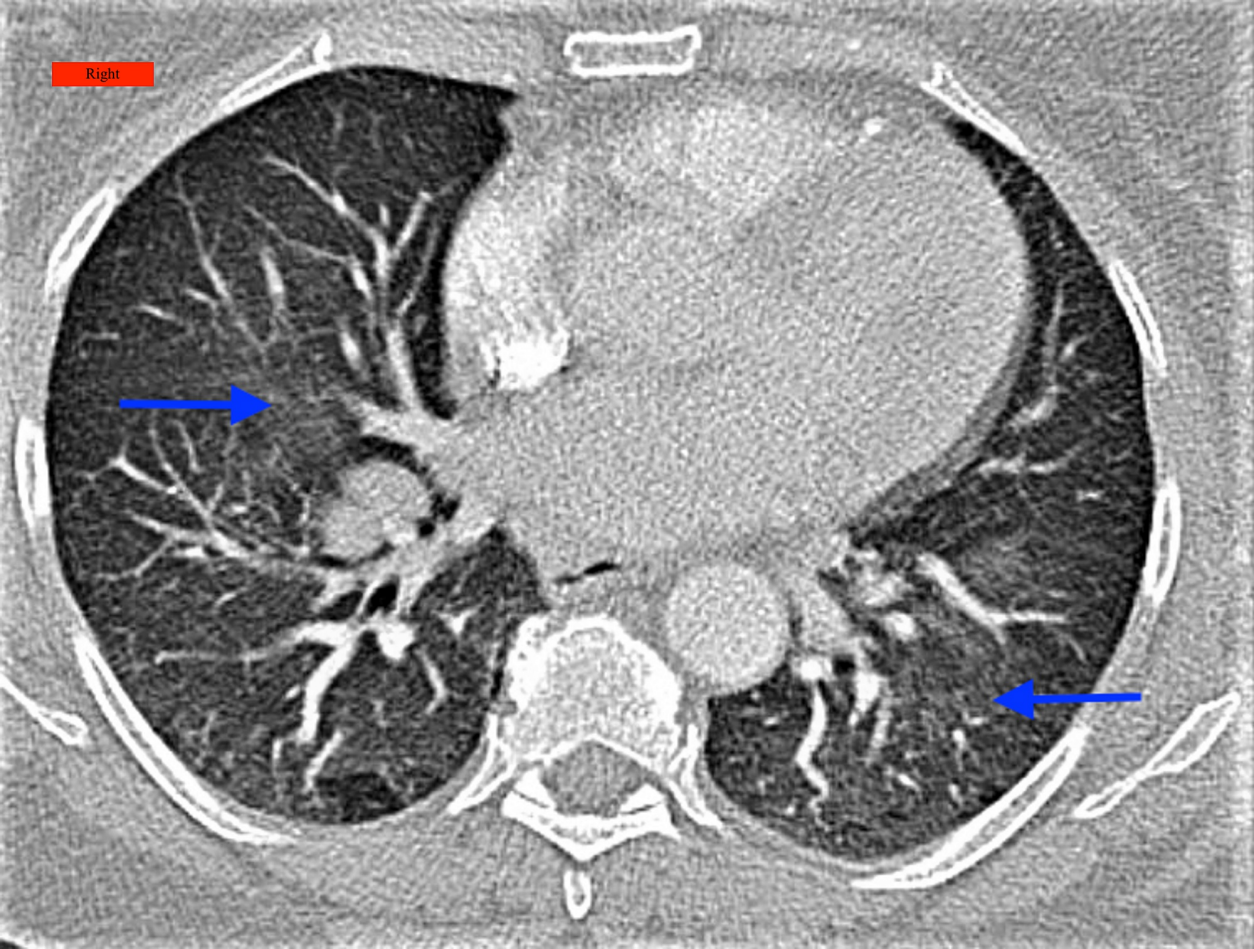
CT chest showed minimal scarring with mild ground-glass opacities in the bilateral lower lobes (arrows) Right: Indicates the right side of the patient; CT: Computed Tomography

A 59-year-old male, with a past medical history of hypertension, diabetes mellitus, asthma, hyperlipidemia, obesity, and obstructive sleep apnea on continuous positive airway pressure (CPAP), presented to the pulmonary clinic with a complaint of shortness of breath for two months. The patient was diagnosed with COVID-19 infection two months ago when he had nasal congestion and rhinorrhea. The patient was treated with steroids and azithromycin by his primary care physician and did not require hospitalization. During the initial visit to the clinic, the patient reported using inhaled budesonide/formoterol and inhaled albuterol as needed. Pulmonary function test revealed restrictive patterns (Table 1) and a CT chest showed minimal scarring with central airways being patent, with mild GGO in the bilateral lower lobes, no pulmonary mass or consolidation, no honeycombing, no pleural effusion, no pneumothorax, and no worrisome pleural thickening (Figure 3). Appropriate counseling was provided to the patient to continue inhalers, CPAP at night, and interval follow-up in six weeks to confirm assess the progress.

Case 4

**Figure 4:**
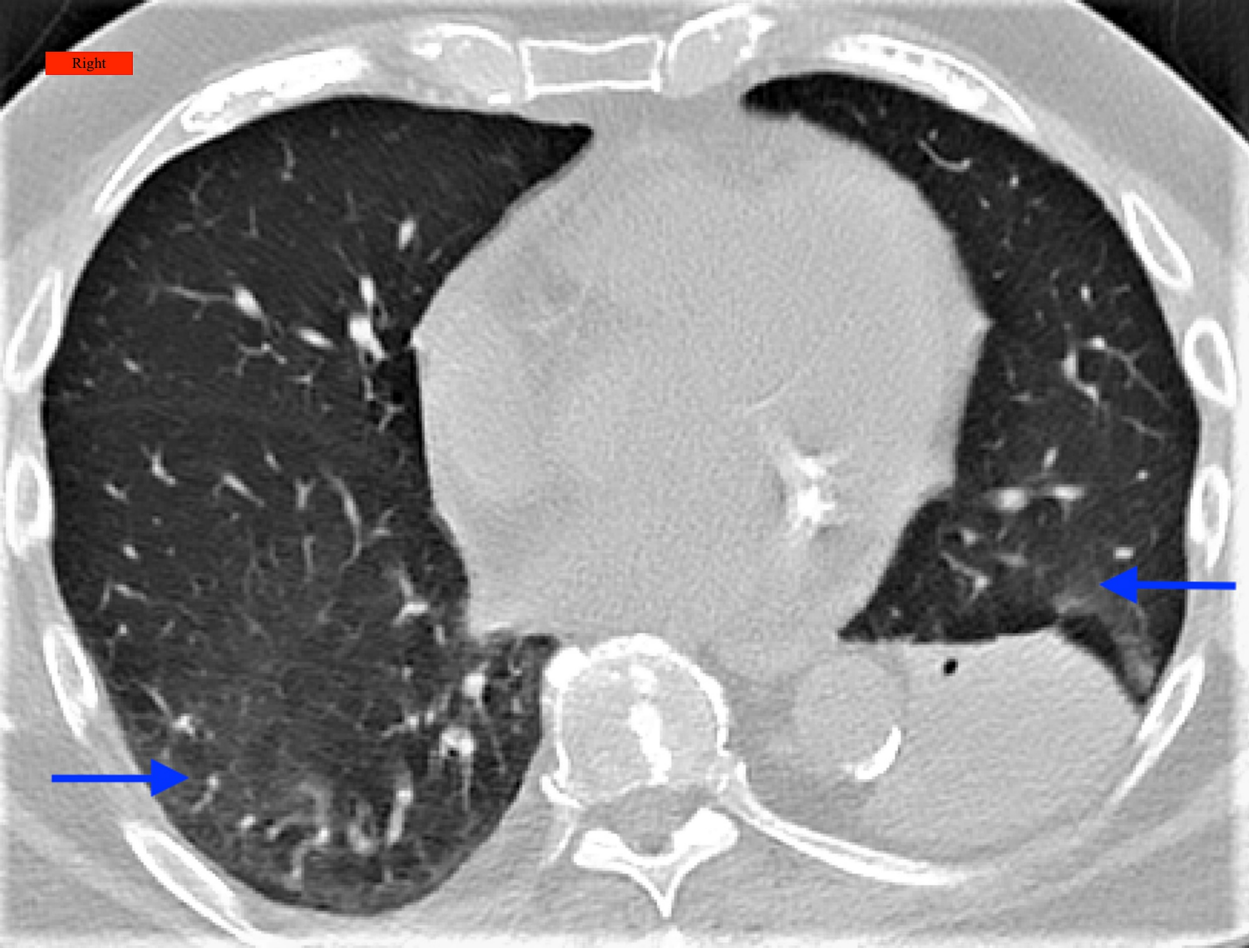
CT chest showing consolidation and volume loss in the left lower lobe and mild ground-glass opacities bilaterally (arrows) Right: Indicates the right side of the patient; CT: Computed Tomography

A 77-year-old male, ex-smoker, with a past medical history of hypertension and hyperlipidemia, presented to the pulmonary clinic with complaints of shortness of breath on exertion, occasional wheezing, and intermittent dry cough. CT chest showed consolidation and volume loss in the left lower lobe and mild GGO bilaterally (Figure 4). The patient was scheduled for bronchoscopy and PFT. Bronchoscopy showed mucous plugging leading to the atelectasis of the left lower lobe and bronchoalveolar lavage (BAL) results were negative for any infection. PFT showed restrictive patterns with a mild reduction in DLCO (Table 1). Upon the follow-up visit in six weeks, the patient’s dyspnea and cough significantly improved. An interval CT scan of the chest was recommended in three months to ensure the resolution of prior findings.

Case 5

**Figure 5:**
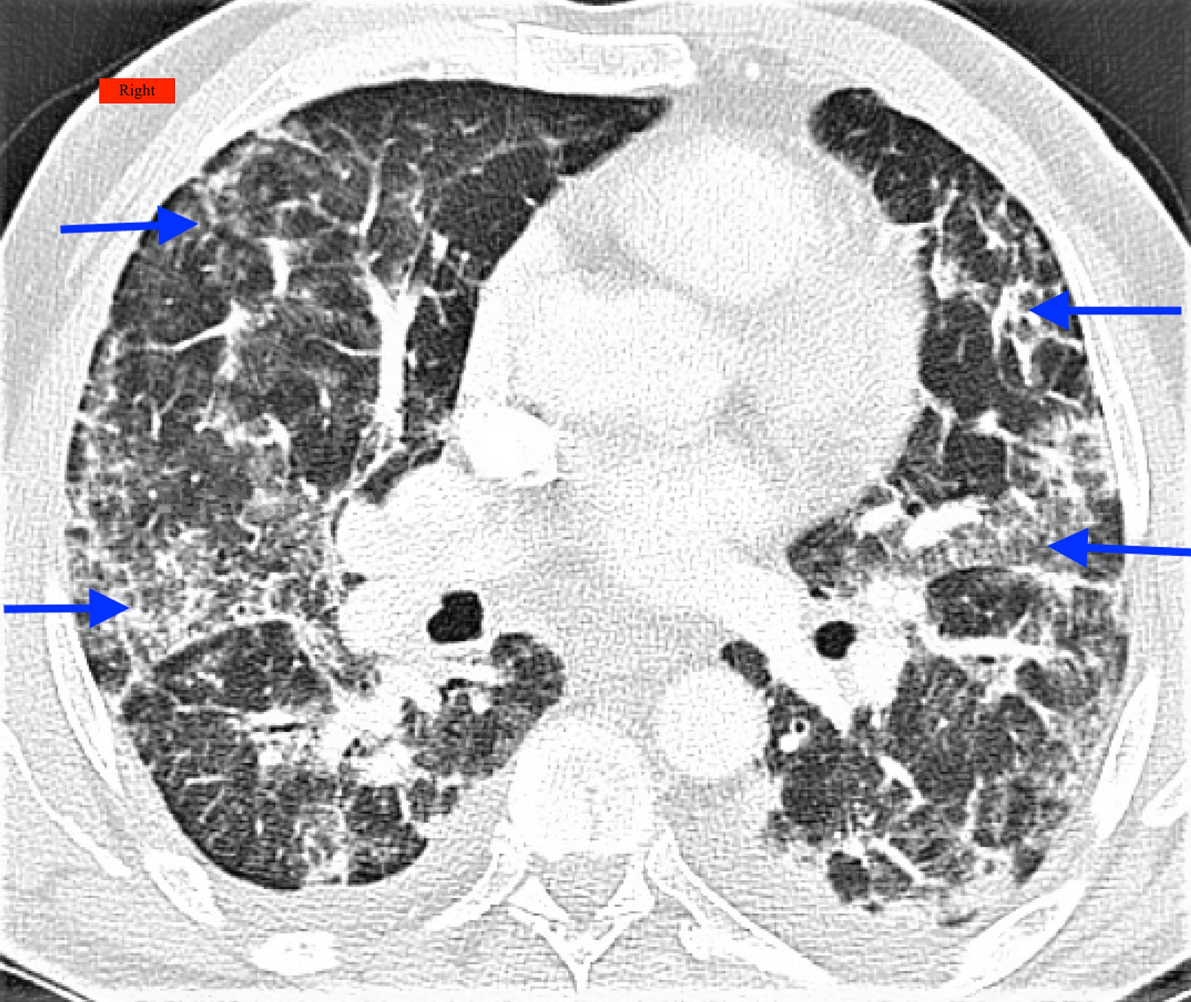
CT scan of the chest showing diffuse interstitial and airspace opacities bilaterally along with small bilateral pleural effusion Right: Indicates the right side of the patient; CT: Computed Tomography

A 57-year-old male, with a past medical history of diabetes mellitus, chronic lymphocytic leukemia, and benign prostatic hyperplasia, was diagnosed with COVID-19 infection three months before visiting us. He just had rhinorrhea and headache and did not require ED or hospital visit. Three months post his diagnosis of COVID-19, he started developing shortness of breath with exertion, which prompted him to follow up with his primary care physician. The patient was again tested for COVID-19 infection for suspicion of reinfection but the test was negative and tested negative for influenza A and B. His shortness of breath continued to worsen to a point he started having difficulty breathing and went to the urgent care where he was found to be saturating at 80% on room air. The patient was placed on oxygen therapy via a nasal cannula and was transferred to the hospital, where he was admitted for acute hypoxic respiratory failure. CT scan of the chest showed diffuse interstitial and airspace opacities bilaterally along with small bilateral pleural effusion suggesting post-COVID fibrosis (Figure 5). Pulmonary function tests could not be obtained. The patient was placed on steroids and antibiotics.

## Discussion

Coronaviruses are single-stranded ribonucleic acid (RNA) viruses that are enveloped [7]. They affect various host species [7]. Based on the genomic structure, coronaviruses can be divided into α, β, γ, and δ but out of these genera, only α and β coronaviruses infect mammals [8]. α coronaviruses, such as 229E and NL63, are some of the known coronaviruses implicated in common cold and croup. Severe acute respiratory syndrome coronavirus (SARS-CoV), Middle East respiratory syndrome coronavirus (MERS-CoV), and SARS-CoV-2, causing the current COVID 19 pandemic, all belong to β coronaviruses [9]. Four main structural proteins of coronavirus are spike, membrane, envelope, and nucleocapsid [10]. Spike protein holds have two main functional subunits. One of those subunits has the ability to bind to the host cell receptor while the other subunit is responsible for the fusion of viral and cellular membranes. Just as in the case of SARS-CoV, the angiotensin-converting enzyme 2 (ACE2) receptor was found to be the main functional receptor for SARS-CoV-2 [11-13]. ACE2 expression is found to be especially high in the lung, heart, kidney, bladder, and ileum [14]. In the lung, ACE2 expression has been found to be maximal on the apical side of the lung epithelial cells in the alveolar space. The SARS-CoV-2 virus can enter the alveolar spaces in the apices and destroy them. This manifests as GGO on CT scan, which can be seen even in asymptomatic patients [15]. Three main cells of the human innate immune apparatus that are known to fight an invading virus are: dendritic cells, alveolar macrophages, and epithelial cells [16]. Dendritic cells and macrophages can phagocytize apoptotic cells infected by the virus, which leads to antigen presentation to the T cells, where the CD4+ T cells activate B cells to promote the production of virus-specific antibodies and CD8+ T cells kill virally infected cells [17]. Patients with severe COVID-19 disease are found to have lymphopenia. There is a reduction in peripheral blood T cells primarily due to exhaustion. This is accompanied by an increase in plasma concentrations of proinflammatory cytokines [18]. Infiltration of all these inflammatory cells consisting of a constellation of innate and adaptive immune cells can augment the fight against the virus but as a side effect can also act as a double-edged sword by inducing lung injury [19-20] resulting in ground-glass opacities on CT scan, which eventually can lead to lung fibrosis. Due to the novelty of coronavirus and its emerging nature, we can only predict and speculate the long-term pulmonary sequelae in patients who have recovered from the virus. It would be difficult to accurately describe the details of pulmonary consequences of COVID-19 in absence of data emanating from a large systematic well-designed study. Our case series is unique in the sense that it discusses the pulmonary sequelae of COVID-19 in patients who recovered with subclinical manifestations, to begin with. Our patient cohort had CT chest findings predominantly suggestive of resolving ground-glass opacities and new pulmonary fibrosis. PFTs were consistent with restrictive lung disease patterns. Patients mainly had symptoms of dyspnea, intermittent cough, and lingering fatigue. Our case series will add to current literature intending to determine long-term pulmonary sequelae of COVID-19. It will hopefully encourage other physicians to look beyond the symptomatic cohort of patients who recovered from COVID-19 and include the subclinical cases in their follow-up plan.

## Conclusions

As the number of patients recovering from COVID-19 continues to soar, pulmonary consequences and its optimal management strategy is still unclear. Emerging data from the larger systematic studies in the future will be able to decipher a great deal of speculative thoughts about pulmonary consequences. Meanwhile, we should continue to be vigilant about patients who develop symptoms due to sequelae of the COVID-19 infection and follow them up at frequent intervals. The establishment of dedicated COVID-19 clinics and utilization of chest radiography, oxygen monitoring, and pulmonary function tests can be handy in monitoring the progress of these patients. It is also imperative that we include recovered patients in our follow up strategy who were asymptomatic or minimally symptomatic at the outset. We might be bracing ourselves for a surge of interstitial lung disease due to the current pandemic and should work briskly to devise diagnostic and management strategies.
